# Predicting the Cytotoxic Potency of Cigarette Smoke by Assessing the Thioredoxin Reductase Inhibitory Capacity of Cigarette Smoke Extract

**DOI:** 10.3390/ijerph13030348

**Published:** 2016-03-21

**Authors:** Longjie Zhang, Min Ning, Yingbo Xu, Chenghui Wang, Guangshan Zhao, Qingqing Cao, Jinsong Zhang

**Affiliations:** 1 Anhui Key Laboratory of Tobacco Chemistry, Technology Center, China Tobacco Anhui Industrial CO., LTD., Hefei 230088, China; chinaren602@126.com (L.Z.); ningminah@aliyun.com (M.N.); xuybah@hotmail.com (Y.X.); wch7664@aliyun.com (C.W.); 2 State Key Laboratory of Tea Plant Biology and Utilization, School of Tea & Food Science, Anhui Agricultural University, Hefei 230036, China; guangshan.Zhao@ahau.edu.cn (G.Z.); qingqing@ahau.edu.cn (Q.C.)

**Keywords:** cigarette smoke, thioredoxin reductase, cytotoxicity, high throughput

## Abstract

The present study investigated the influence of the cigarette smoke extract (CSE) on mammalian thioredoxin reductase (TrxR) activity. TrxR is a selenoenzyme with a selenocysteine (Sec) residue exposed on the enzyme’s surface. This unique Sec residue is particularly susceptible to modification by numerous types of electrophiles, leading to inactivation of TrxR and consequent cytotoxicity. Cigarette smoke contains various electrophiles, and the present study showed that CSE could inhibit intracellular TrxR through causing crosslinking and alkylation of TrxR1. TrxR inhibitory capacities of various CSEs were evaluated by using mouse-liver homogenate. Among the CSEs prepared from 18 commercial cigarette brands, TrxR inhibitory capacities of the maximum and the minimum had a 2.5-fold difference. Importantly, CSE’s inhibitory capacity greatly paralleled its cytotoxic potency in all cell lines used. Compared to cytotoxic assays, which have been widely used for evaluating cigarette toxicity but are not suitable for simultaneously examining a large number of cigarette samples, the present method was simple and rapid with a high-throughput feature and thus could be used as an auxiliary means to predict the cytotoxicity of a large number of cigarette samples, making it possible to extensively screen numerous agricultural and industrial measures that potentially affect cigarette safety.

## 1. Introduction

Cigarette smoking has been recognized a major risk factor for many illnesses, including cardiovascular diseases, cancers, and respiratory diseases [1]. Cigarette smokers are always advised to give up smoking; however, there are more than 1 billion individuals worldwide who are reluctant or unable to successfully quit. The United States National Academy of Medicine has indicated that reducing the risk of disease by reducing exposure to tobacco toxicants is feasible [2]; thus, cigarette manufacturers should provide less harmful cigarettes—so-called potentially reduced exposure products (PREPs)—for smokers.

Cigarette smoke formed from the pyrolysis of the tobacco leaf is a dynamic and complex aerosol comprising more than 5600 chemical constituents [3], of which 158 compounds are health hazards [4]. Numerous factors, such as added ingredients, threshing and re-drying, storage and fermentation, reconstitution and blend, tobacco varieties and culture conditions, and cigarette filter materials and devices, could influence the levels and constituents of the hazardous compounds present in the inhaled complex mixtures [5,6]. To develop and refine PREPs, all available agricultural and industrial measures that potentially affect cigarette toxicity should be carefully investigated. *In vitro* cytotoxic assays, which are a major part of a series of short-term toxicological evaluations, have been widely used [6]; however, this evaluation modality is not suitable for an in-depth and robust examination of numerous factors because it can simultaneously evaluate only a limited number of samples. For example, each sample must be tested at six proper concentrations with six replicates to calculate the half maximal inhibitory concentration (IC_50_) because of the non-linear concentration-dependent feature of the cytotoxic action and greater variation in cell viability. To compare 10 samples with three replicates each, 12 96-well plates are needed, and each procedure performed in that many wells is an uncommon and prone to error; therefore, a high-throughput method for screening a large number of samples is needed.

A thioredoxin (Trx) system comprising Trx, Trx reductase (TrxR), and nicotinamide adenine dinucleotide phosphate (NADPH) plays an important role in promoting cell proliferation, maintaining redox homeostasis, and counteracting apoptosis. TrxR, with a selenocysteine (Sec) residue at the C-terminal domain of the enzyme, is a well-documented selenoprotein [7]. Sec residue, which exposes the surface of TrxR and maintains full ionization at physiological pH because of its unusually low 5.2 pKa, is essential for sustaining TrxR maximum activity [8]. TrxR, because of its unusually high nucleophilicity and highly accessible Sec residue, is particularly susceptible to inactivation by various electrophilic compounds, such as quinones and unsaturated aldehydes [9,10,11]. Cigarette smoke contains several electrophilic compounds, including quinones and α, β-unsaturated aldehydes [12,13,14]; therefore, it was hypothesized that cigarette smoke can target TrxR. Using mouse-liver homogenate, CSE was observed to inactivate TrxR. In addition, TrxR inhibitory capacity of CSE greatly paralleled its cytotoxic potency. Thus, this simple, rapid, and accurate method with a high-throughput feature can be used to predict the cytotoxicity of a large number of cigarette samples.

## 2. Materials and Methods

### 2.1. Chemicals

NADPH, 5,5-dithiobis (2-nitrobenzotic acid) (DTNB), glutathione reductase (GR, from *Escherichia coli*), dimethylsulfoxide (DMSO), 3-(4,5-dimethyl-2-thiazolyl)-2,5-diphenyltetrazolium bromide (MTT), bovine serum albumin, and digitonin were obtained from Sigma-Aldrich Corporation (St. Louis, MO, USA). Auranofin was purchased from Santa Cruz Biotechnology, Inc. (Santa Cruz, CA, USA). Trypsin, penicillin, streptomycin, RPMI 1640 medium, and fetal calf serum were products of HyClone (Logan City, UT, USA). Biotin hydrazide, sodium cyanoborohydride (NaCNBH_4_), spin desalting columns, centrifuge columns, and streptavidin agarose resin were obtained from Thermo Scientific (Waltham, MA, USA). Other chemicals were of the highest grade available. Cigarettes were purchased from a market.

### 2.2. Cell Lines

The human squamous carcinoma cell line (Tca8113) was obtained from the Key Laboratory of Oral Biomedicine of Shanghai Jiaotong University, China. The murine colon carcinoma cell line (CT26) and human liver hepatocellular carcinoma cell line (HepG2) were purchased from ATCC (Manassas, VA, USA). The RAT-vascular smooth muscle cell (VSMC) was provided by Zhongwen Xie of State Key Laboratory of Tea Plant Biology and Utilization, Anhui Agricultural University, Anhui, China.

### 2.3. Preparation of CSE

**Figure 1:**
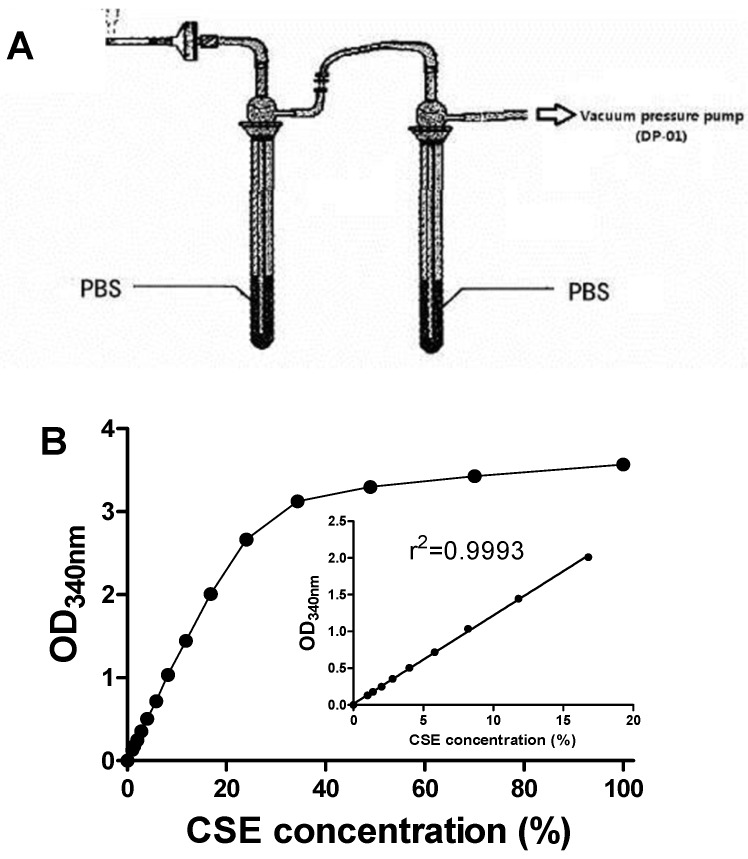
Device for preparing cigarette smoke extract (CSE) (**A**) and assessment of CSE concentration (**B**). Data are presented as means ± SEM (*n* = 2).

CSE was prepared as described elsewhere [15] with a slight modification. Briefly, two gas-absorption bottles containing a 5-mL potassium phosphate buffer (PBS, 100 mM, pH 7.0 with 10 mM EDTA-Na_2_) in each bottle were linked. The DP-01 vacuum pressure pump (Shanghai Mosu Scientific Equipment Co., Ltd., Shanghai, China) with a constant air flow rate of 10 L/min was used to provide a sufficient suction force so that mainstream smoke from one cigarette (unless otherwise specified) was rapidly and continuously bubbled into 10-mL PBS (Figure 1A). The smoke delivery length of one cigarette was maintained at approximately five-sixths of the total length to avoid confounding combustion of the filter. The resultant dark-yellow solution was passed through a 0.22-μm micropore filter to remove particulates and immediately stored at −80 °C. CSE concentration of the collected solution was measured by the optical density at a wavelength of 340 nm (Figure 1B) [15,16,17]. OD_340 nm_ normalization was used to eliminate the influence of delivery length.

### 2.4. Cell Culture and Viability Assay

Cells were maintained at 37 °C under 5% CO_2_ and 95% air in the complete medium (RPMI-1640 medium supplemented with 10% (*v*/*v*) fetal calf serum, 100 U/mL penicillin, and 100 μg/mL streptomycin). Cells were seeded at a density of 1 × 10^5^ cells/well in a 96-well cell culture plate and were allowed to attach for 24 h. Cells were then treated with CSE dissolved in the complete medium for 24 h. After removing the medium, a 200-μL complete medium containing 0.5 mg/mL MTT were added to each well. After incubation for 4 h, the medium was replaced by 150 μL of DMSO, and the absorbance at 490 nm was measured.

### 2.5. Preparation of Cell Lysate

Tca8113 cells were cultured in 6-well plates or 10-cm dishes to reach 90% confluence. Following CSE treatments, cells were lysed in 0.1% digitonin dissolved in 0.1 M Tris-HCl (pH 7.4) via ultrasonic wave for 20 min in an ice-bath. The cell lysates were centrifugated at 10,000 rpm and 4 °C for 10 min, and the resultant supernatant was stored at −80 °C. Protein was quantified using the BCA protein assay kit (Pierce).

### 2.6. Separation of Alkylated Proteins

Cell lysate (>20 mg protein/mL) was mixed with 5 mM of biotin hydrazide dissolved in DMSO at room temperature for 2 h with constant rotation. After the addition of 30 mM of NaCNBH_4_, the mixture was incubated on ice for 60 min. Excess biotin hydrazide and NaCNBH_4_ were removed by spin desalting columns. Biotin-labeled proteins were then separated by streptavidin agarose resin. Proteins adhere to the resin were eluted with a reducing 5×SDS-PAGE loading buffer.

### 2.7. Western Blotting

Samples were boiled in 5×SDS-PAGE loading buffer at 95 °C for 10 min, and then the resultant samples were loaded onto a 12% SDS-PAGE gel for electrophoresis. Proteins were transferred to a PVDF membrane. The membrane was blocked with 5% nonfat dried milk in TBS-T (10 mM Tris-HCl, pH 7.8, 150 mM NaCl, and 0.05% (*v*/*v*) Tween-20) for 2 h at room temperature. After overnight incubation with a polyclonal antibody raised against the mouse TrxR1 protein (prepared by Dr. Gary, F. Merrill, Department of Biochemistry and Biophysics, Oregon State University), which was diluted in TBS-T (1:8000) at 4 °C, the membrane was washed twice with TBS-T for 10 min and once with TBS for 10 min, and then incubated with a secondary HRP-conjugated antibody diluted in TBS-T (1:8000) for 1 h at room temperature. The membrane was washed twice with TBS-T for 10 min and once with TBS for 10 min. Proteins in the membrane were detected using the ChemiDoc XRS+ detection system (ECL, Bio-Rad, Hercules, CA, USA).

### 2.8. Preparation of Mouse-Liver Homogenate

Liver tissues were excised from healthy male Kunming mice after the mice were euthanized, rinsed in ice-cold saline, and homogenized in ice-cold PBS (150 mM, pH 7.2 with 1 mM EDTA-Na_2_) using a high-throughput tissue lyser (1:9 *w*/*v*). After centrifugation at 10,000 *g* and 4 °C for 20 min, the supernatant was immediately stored at −80 °C. All protocols for the animal experiments complied with the guidelines of Anhui Agricultural University for care and use of laboratory animals.

### 2.9. TrxR Inhibitory Capacity of CSE

Mouse-liver homogenate and CSE or PBS (100 mM, pH 7.0 with 1 mM EDTA-Na_2_) were incubated in the presence of 480 µM of NADPH at 37 °C for 20 min. Then, TrxR activity was assessed utilizing NADPH-dependent DTNB reduction, which is inhibited by auranofin according to the method of Smith and Levander [18] with some modifications [19]. Briefly, a stock mixture (10 mM EDTA-Na_2_, 5 mM DTNB, 240 µM NADPH, and 0.2 mg/mL BSA in 100 mM PBS, pH 7.4) was prepared and kept at 37 °C before the assay. Samples were mixed with 1.47 mM auranofin containing 5% ethanol or 5% ethanol at a ratio of 9:1 (*v*:*v*) at 37 °C for 10 min to prepare paired samples for the measurement of activity that was reduced by auranofin, a highly specific inhibitor of TrxR. The samples were mixed with 250 μL of stock mixture in a well of a 96-well plate and changes in absorbance at 412 nm were monitored at 37 °C by a microplate reader. TrxR activity was calculated by subtracting the rate of the reaction with auranofin from the rate of the reaction without auranofin. One unit of TrxR activity was defined as 1 μmol of NADPH oxidized/min/mg protein. TrxR inhibitory capacity of CSE was normalized by CSE concentration.

### 2.10. Assays of Other Enzymatic Activities

Glutathione peroxidase (GPx) was determined using the method of Smith and Levander [18], and the activity was calculated in terms of μmols NADPH oxidized/min/mg protein. GR activity was measured using the method of Carlberg and Mannervik [20] and was calculated in terms of nmols NADPH oxidized/min/mg protein. Superoxide dismutase (SOD) activity was estimated using xanthine/xanthine oxidase and nitroblue tetrazolium [21]. One unit of SOD activity was defined as the amount of protein that inhibited the rate of nitroblue tetrazolium reduction by 50%. Data were expressed as U/mg protein. Catalase (CAT) activity was assayed on the basis of its ability to decompose H_2_O_2_, which was measured at 240 nm [22]. One unit of CAT activity was defined as nmol H_2_O_2_ consumed/min/mg protein. Glutathione S-transferase (GST) activity was chemically determined using 1-chloro-2,4-dinitrobenzene (CDNB) [23]. One unit of GST activity was calculated in terms of nmols CDNB changed/min/mg protein.

### 2.11. Statistical Analyses

Data were presented as the mean ± SEM. The differences between groups were examined by one way analysis of variance *post hoc* Tukey’s multiple comparison tests or Student’s test as appropriate using GraphPad (Prism version 5, San Diego, CA, USA). A *p* value of <0.05 was considered statistically significant.

## 3. Results

### 3.1. Influence of CSE on the Activities of Antioxidant Enzymes

**Figure 2:**
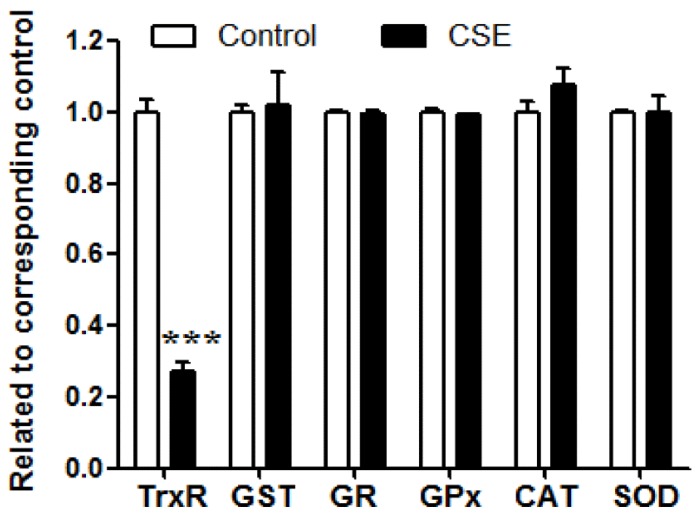
Effects of cigarette smoke extract (CSE) on antioxidant enzyme activities of mouse-liver homogenate. CSE (80 μL) was mixed with mouse-liver homogenate (50 µL) for 20 min at 37 °C. Data are presented as means ± SEM (*n* = 3), compared to control, *** *p* < 0.001. Basal average activities of thioredoxin reductase (TrxR), glutathione S-transferase (GST), glutathione reductase (GR), glutathione peroxidase (GPx), catalase (CAT), and superoxide dismutase (SOD) were 3.2 mU/mg protein, 0.2 U/mg protein, 46.5 U/mg protein, 60.5 mU/mg protein, 19.5 U/mg protein, and 6.3 U/mg protein, respectively.

After CSE and mouse-liver homogenate were incubated for 20 min at 37 °C, CSE inhibited TrxR activity by 73% (*p* < 0.001); however, it did not significantly alter the activities of GST, GR, GPx, CAT, or SOD (Figure 2). CSE showed a priority characteristic of inhibiting TrxR activity among the investigated antioxidant enzymes.

### 3.2. Time and Dose Effects of CSE on Trxr Activity

**Figure 3:**
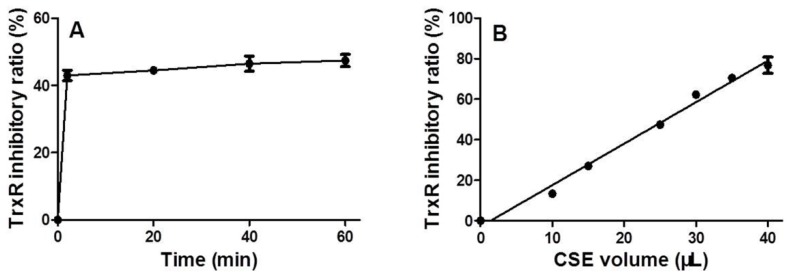
Time (**A**) and dose (**B**) effects of cigarette smoke extract (CSE) on thioredoxin reductase (TrxR) activity. Data are presented as means ± SEM (*n* = 2).

After CSE and mouse-liver homogenate were incubated for specific time periods at 37 °C, CSE immediately inhibited TrxR activity; thereafter, there was no significant increase in the inhibition ratio with an increase in time (Figure 3A). Using a reaction time of 20 min, CSE inhibited TrxR activity in a dose-dependent manner with a Pearson correlation coefficient as high as 0.9953 (*p* < 0.0001) (Figure 3B).

### 3.3. Comparison of TrxR Inhibitory Capacity of CSEs Prepared from Different Cigarette Brands

**Figure 4:**
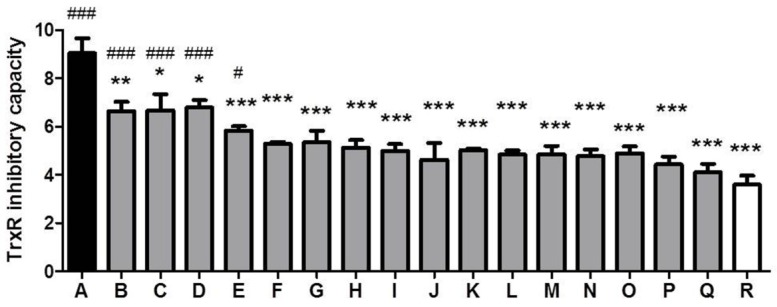
Comparison of thioredoxin reductase (TrxR) inhibitory capacity of different cigarette brands. Data are presented as means ± SEM (*n* = 3). Compared to brand A, * *p* < 0.05; ** *p* < 0.01; *** *p* < 0.001. Compared to brand R, ^#^ *p* < 0.05; ^###^ *p* < 0.001.

Eighteen cigarette brands were purchased from a market to compare their TrxR inhibitory capacities. Three cigarettes from each brand were used to prepare three CSE replicates. TrxR inhibitory capacities of the maximum and the minimum had a 2.5-fold difference (*p* < 0.001) (Figure 4).

### 3.4. Cytotoxic Potency of CSEs with Different TrxR Inhibitory Capacities

**Figure 5:**
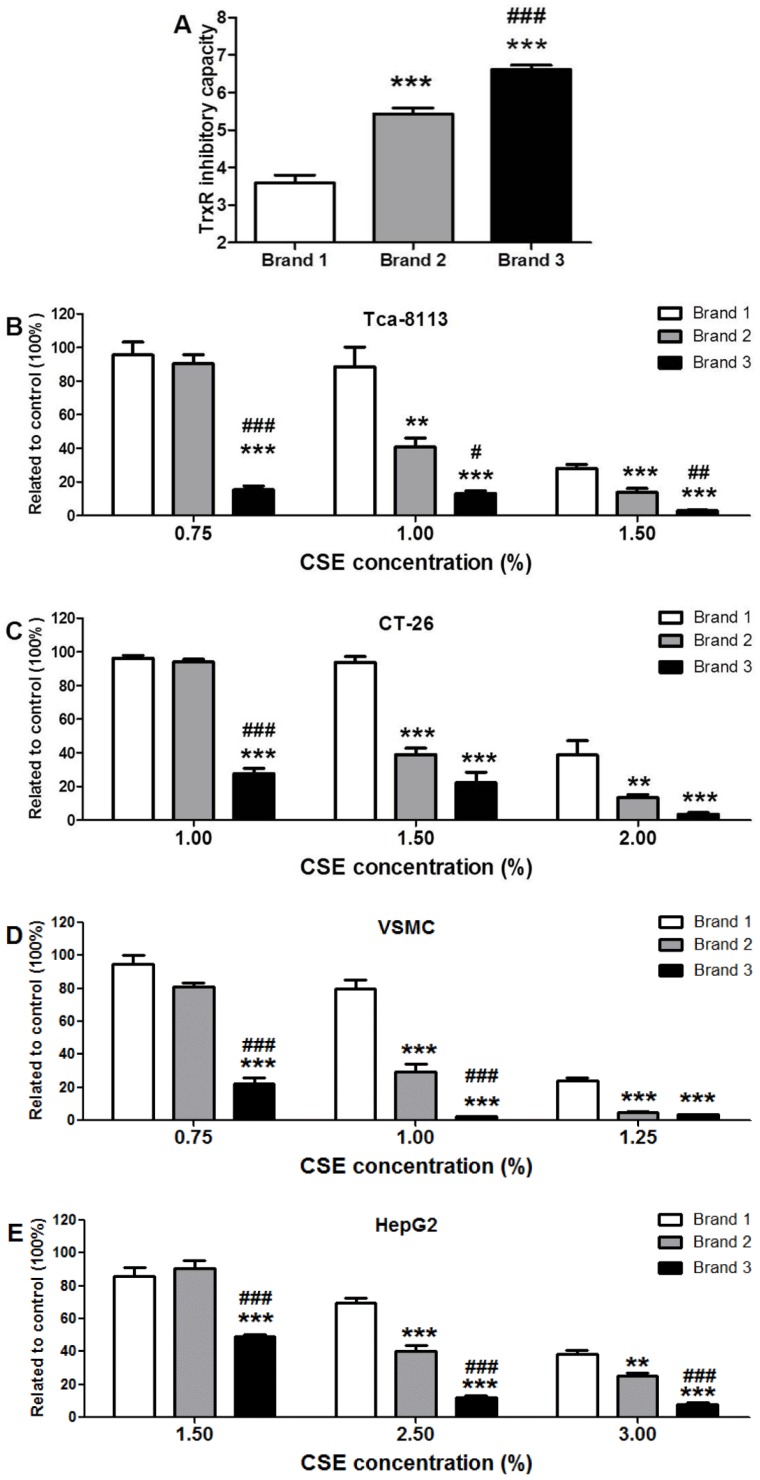
Cytotoxic evaluations of cigarette smoke extracts (CSEs) with different thioredoxin reductase (TrxR) inhibitory capacities; (**A**) TrxR inhibitory capacity of CSEs prepared from 3 commercial brands; (**B**–**E**) cell viability of indicated cell lines. Data are presented as means ± SEM (*n* = 6). Compared to Brand 1, ** *p* < 0.01; *** *p* < 0.001. Compared to Brand 2, ^#^ *p* < 0.05; ^##^ *p* < 0.01; ^###^ *p* < 0.001.

Three cigarette brands were used for measuring their capacities to inhibit TrxR and their cytotoxic activities. To maintain the added CSE solution below 3.0% in the cell culture medium to eliminate the influence of diluted medium on cell survival and to ensure a solid cytotoxicity during the 24-h cell culture, concentrated CSE was prepared using five cigarettes. As shown in Figure 5A, the capacities of concentrated CSEs prepared from the three brands to inhibit TrxR were significantly different. Specifically, the capacity of Brand 3 to inhibit TrxR was 84% higher than that of Brand 1 (*p* < 0.001) and 22% higher than that of Brand 2 (*p* < 0.001); the capacity of Brand 2 to inhibit TrxR was 52% higher than that of Brand 1 (*p* < 0.001).

To compare cytotoxicity, the concentrated CSEs were normalized by OD_340 nm_ and added to the cell culture medium at the indicated concentrations. Although the examined cell lines were not unanimously sensitive to a specific CSE, likely due to different resistance background, all CSEs generated a dose-dependent cytotoxic effect (Figure 5B–E). Brand 3, with the maximum capacity for inhibiting TrxR, was always more toxic than Brand 1, which had the minimum capacity for inhibiting TrxR, at all tested concentrations (*p* all <0.001). At the two higher concentrations, Brand 2 was more toxic than Brand 1 (*p* < 0.001 or 0.01). At the lowest concentration, Brand 3 was more toxic than Brand 2 (*p* all <0.001). Taken together, these results likely indicate that CSE’s capacity to inhibit TrxR may be correlated with its cytotoxic activity.

### 3.5. CSE Inhibits Intracellular TrxR Activity and Causes Crosslinking and Alkylation of Intracellular TrxR1

**Figure 6:**
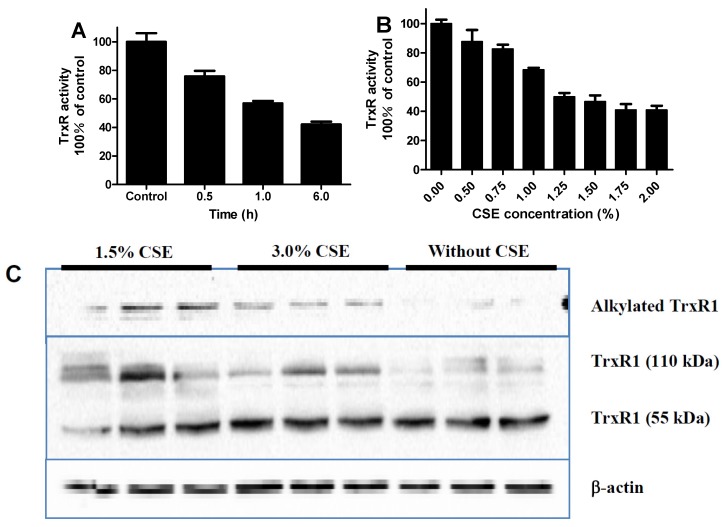
Impact of cigarette smoke extract (CSE) on intracellular TrxR; (**A**) time-dependent alteration of TrxR activity in Tca-8113 cells treated with 1.5% CSE; (**B**) dose-dependent alteration of TrxR activity in Tca-8113 cells treated with CSE for 1 h; (**C**) TrxR1 protein in Tca-8113 cells treated with CSE for 2 h. Basal TrxR activity was 10.1 and 10.4 (mU/mg protein) in A and B, respectively. Data are presented as means ± SEM (*n* = 2 in A and 3 in B and C).

In Tca-8133 cells, CSE inhibited intracellular TrxR activity in a time-dependent manner at a concentration of 1.5% (Figure 6A) and in a concentration-dependent fashion at 1 h (Figure 6B). CSE at the concentrations of 1.5% and 3.0% resulted in crosslinking and alkylation of intracellular TrxR1 at 2 h, as indicated by the increase of 110 kDa TrxR1 and biotin-labeled TrxR1, respectively (Figure 6C).

## 4. Discussion

The present study showed that CSE could inhibit intracellular TrxR, leading to crosslinking and alkylation of TrxR1 (Figure 6), and CSE also rapidly inhibited TrxR activity in mouse-liver homogenate (Figure 3A). It can be anticipated that a multitude of noxious compounds in CSE collectively inhibit TrxR activity. Peroxynitrite, an active principle in CSE [24,25], can inhibit TrxR [26] presumably through TrxR nitration [27]. Acrolein, an α,β-unsaturated aldehyde in CSE, is a potent TrxR inhibitor [15]. CSE contains hydroquinones, quinones, and semiquinone radicals, particularly, o- and p-benzosemiquinones [12,13], whereas this class of compounds invariably has a strong TrxR inhibitory capacity [9,10,11]. Chromium, mercury, and arsenic compounds have been detected in cigarette smoke [28,29], and these metal and metalloid compounds can also inhibit TrxR activity [30,31,32,33]. Harmful low molecular–weight carbonyl compounds, such as dicarbonyls including glyoxal and methylglyoxal, are formed in cigarette smoke [34]. Glyoxal and methylglyoxal have been demonstrated to inactivate GPx activity through the electrophilic attack of Sec residue [35]; therefore, it is believed that glyoxal and methylglyoxal would preferentially inhibit TrxR at low concentrations according to the present results (Figure 2). Glutathione-crotonaldehyde adducts have been identified from cells exposed to cigarette smoke [36]; thus, it is conceivable that crotonaldehyde, a highly reactive α,β-unsaturated aldehyde and a major harmful compound found in CSE, would also inactivate TrxR because Sec residue in selenoproteins can react 10^7^ times faster with electrophiles than free cysteine [37]. It has been reported that methyl vinyl ketone and 2-cyclopenten-1-one participate in the cytotoxic action of CSE [38]; therefore, it was conjectured that these electrophilic compounds would also target TrxR. With regard to glyoxal, methylglyoxal, crotonaldehyde, methyl vinyl ketone, and 2-cyclopenten-1-one, additional studies are warranted to confirm their TrxR inhibitory capacities; however, it would be difficult to figure out TrxR inhibitory capacities of CSE by detecting these chemicals because each TrxR inhibitory agent in CSE is likely to have a different inhibitory potency, the constituents of these compounds in CSE are complex, and certain inhibitory agents might act synergistically.

In parallel with TrxR, the activities of other antioxidant enzymes in mouse-liver homogenate were also estimated. Although TrxR activity was dramatically reduced, the activities of other examined antioxidant enzymes remained unchanged (Figure 2), demonstrating that the TrxR inhibitory capacity is greater than its capacity to inhibit the activity of other antioxidant enzymes, including GPx, another Sec-containing enzyme. This preferential characteristic is largely attributed to the Sec residue of TrxR. Sec residue in selenoproteins has unusually high nucleophilicity and thus exhibits an exceptionally strong reaction rate with electrophilic compounds [38]. Penultimate Sec residue in TrxR is exposed on the enzyme’s surface, which further enhances its susceptibility to electrophilic attack [8], whereas Sec residue, the active site of GPx, is buried deep inside the enzyme, making it less susceptible than TrxR to electrophilic attack [39].

Although the cytotoxicity of cigarette smoke has been extensively examined, the cytotoxic mechanism of CSE has not been well defined. CSE’s inhibition of TrxR might be one route by which to trigger cytotoxic responses. TrxR is a specific enzyme responsible for catalyzing the reduction of Trx’s active disulfide site using NADPH. The reduced Trx maintained by TrxR can inhibit apoptosis by binding apoptosis signal-regulating kinase 1, sustain cellular proliferation by providing reduction equivalents for ribonucleotide reductase, participate in defense against oxidative stress by donating electrons to peroxiredoxins and methionine sulfoxide reductases, and regulate redox-sensitive transcription factors by reducing their conserved cysteine residues that are required for DNA-binding; therefore, TrxR, as a vital enzyme for Trx, is implicated in regulating a plethora of biological functions, including cell survival [7,11,40]. Derivatization by electrophilic compounds not only inactivates TrxR but also often transforms TrxR from a cell guardian to a cell killer, namely, the resultant Sec thioredoxin reductase-derived apoptotic proteins (SecTRAPs) with NADPH oxidase activity; therefore, the pronounced cytotoxic property of certain TrxR inhibitors is most likely attributed to a synergistic effect of impaired TrxR/Trx functions and the detrimental effects of SecTRAPs on cell survival [11]. Electrophilic mechlorethamine can inhibit cytosolic TrxR activity along with increases of crosslinked TrxR1, such an inactivated TrxR could in turn generate reactive oxygen species [41]. Formation of covalently linked TrxR1 subunits is closely associated with cell death and excessive oxidative stress [42]. The present study showed that CSE also had a similar action like mechlorethamine (Figure 6). Using mouse-liver homogenate, we found that the TrxR inhibitory capacities of CSEs prepared from certain commercial brands of cigarettes are significantly different with a 2.5-fold contrast between the maximum and the minimum (Figure 4). More importantly, TrxR inhibitory capacities of CSEs were greatly parallel to its cytotoxic activities in all examined cell lines (Figure 5), suggesting that (1) CSE’s effect on TrxR might be one mechanism among many that may be responsible for CSE-triggered cell death; and (2) the TrxR inhibitory capacities of CSEs might be sufficiently correlated with cell death as to be useful in the capacity of a predictive assay of cytotoxic activity. Because the measurement of TrxR inhibitory capacity involved only the incubation of CSE and mouse-liver homogenate in the presence of sufficient NADPH and the subsequent kinetic analyses of TrxR activity after adding a working solution comprising DTNB and NADPH, these two procedures could be performed within 30 min using a microtiter plate. Because TrxR inhibitory capacity of CSE was strictly dose-dependent with a Pearson correlation coefficient as high as 0.9953 (*p* < 0.0001) (Figure 3B), only an appropriate CSE concentration is required for measuring its TrxR inhibitory capacity; therefore, in a 96-well plate, 30 different CSEs with three replicates each could be simultaneously compared within a few hours. In contrast, the comparison of 10 different CSEs with three replicates each by cytotoxic methods, such as a MTT assay, should be done over several days using 12 96-well plates according to the aforementioned experimental design. Notably, this simple, rapid, and accurate high-throughput approach makes extensive screening of numerous agricultural and industrial measures that potentially affect cigarette cytotoxicity feasible. It is worth noting that this high-throughput approach is unable to replace the cytotoxicy method, which is actually more sensitive as seen from the comparison of Brand 2 and Brand 3 in Figure 5. Specifically, TrxR inhibitory capacities of these two brands had only a 22% difference albeit significant (*p* < 0.001) (Figure 5A). Therefore, an effective strategy of developing PREPs is the combination of the high-throughput approach and cytotoxicity evaluation; the former could help to find big differences in TrxR inhibitory capacities and accordingly huge differences in cytotoxic activities from numerous samples, while the latter could allow for the identifying of samples with lower cytotoxicity from several samples with lower but similar TrxR inhibitory capacities.

## 5. Conclusions

CSE can inhibit TrxR activity. TrxR inhibitory capacities of CSEs prepared from certain commercial brands of cigarettes were significantly different. A high TrxR inhibitory capacity indicated a strong cytotoxic potency, suggesting that TrxR inhibitory capacity of CSE, which can be rapidly and precisely measured, could be used as an auxiliary screening method by which to predict the cytotoxicity of a large number of cigarette samples.

## Abbreviations

The following abbreviations are used in this manuscript:

CSE: cigarette smoke extract
TrxR: thioredoxin reductase
Sec: selenocysteine
PREPs: potentially reduced exposure products
IC_50_: half maximal inhibitory concentration
Trx: thioredoxin
NADPH: nicotinamide adenine dinucleotide phosphate
DTNB: 5,5-dithiobis (2-nitrobenzotic acid)
GR: glutathione reductase
DMSO: dimethylsulfoxide
MTT: 3-(4,5-dimethyl-2-thiazolyl)-2,5-diphenyltetrazolium bromide
Tca8113: human squamous carcinoma cell line
CT26: murine colon carcinoma cell line
HepG2: human liver hepatocellular carcinoma cell line
VSMC: RAT-vascular smooth muscle cell
PBS: potassium phosphate buffer
NaCNBH_4_: sodium cyanoborohydride
GPx: glutathione peroxidase
SOD: superoxide dismutase
CAT: catalase
GST: glutathione S-transferase
CDNB: 1-chloro-2,4-dinitrobenzene
SecTRAPs: Sec thioredoxin reductase-derived apoptotic proteins
